# (3,5-Dimethyl­pyrazol-1-yl)[5-(3,5-dimethyl­pyrazol-1-ylcarbon­yl)-2-thien­yl]methanone

**DOI:** 10.1107/S1600536809040756

**Published:** 2009-10-17

**Authors:** Ilia A. Guzei, Lara C. Spencer, Mmboneni G. Tshivashe, James Darkwa

**Affiliations:** a Department of Chemistry, University of Wisconsin-Madison, 1101 University Ave, Madison, WI 53706, USA; b Department of Chemistry, University of Johannesburg, Auckland Park Kingsway Campus, Johannesburg 2006, South Africa

## Abstract

The title compound, C_16_H_16_N_4_O_2_S, crystallizes with two symmetry-independent half-mol­ecules in the asymmetric unit. All non-H atoms in each molecule lie in a crystallographic mirror plane. The mol­ecules form sheets in the *ac* plane, which then form stacks along the *b* axis. The sheets are connected *via* π–π stacking inter­actions [centroid–centroid distance between pyrazolato rings = 3.6949 (8) Å].

## Related literature

In the course of our studies toward effective polymerization catalysts we have investigated Pd complexes with pyrazolyl derivatives as ligands, see: Guzei *et al.* (2003 ▶); Mohlala *et al.* (2005 ▶). The title compound was isolated serendipitously during this work. For a description of the Cambridge Structural Database, see: Allen (2002 ▶) and for *Mogul*, see: Bruno *et al.* (2002 ▶). For thiophene carbonyl linker pyrazolyl  compounds,  see: Ojwach *et al.* (2005 ▶).
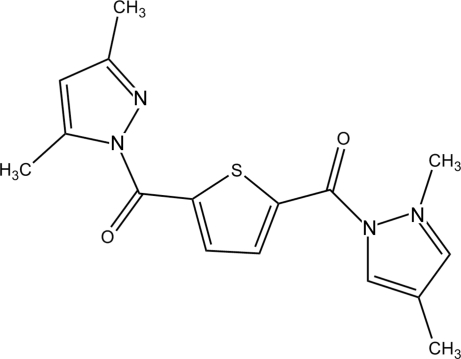


## Experimental

### 

#### Crystal data

C_16_H_16_N_4_O_2_S
*M* *_r_* = 328.39
Monoclinic, 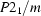

*a* = 15.615 (3) Å
*b* = 6.7153 (16) Å
*c* = 16.803 (4) Å
β = 114.452 (4)°
*V* = 1603.9 (6) Å^3^
*Z* = 4
Mo *K*α radiation
μ = 0.22 mm^−1^
*T* = 296 K
0.30 × 0.30 × 0.20 mm

#### Data collection

Bruker CCD 1000 area-detector diffractometer
Absorption correction: multi-scan (*SADABS*; Bruker, 2007 ▶) *T*_min_ = 0.938, *T*_max_ = 0.958
7557 measured reflections
3297 independent reflections
2744 reflections with *I* > 2σ(*I*)
*R*_int_ = 0.017

#### Refinement

*R*[*F* ^2^ > 2σ(*F* ^2^)] = 0.039
*wR*(*F* ^2^) = 0.112
*S* = 1.03
3297 reflections
286 parameters
H-atom parameters constrained
Δρ_max_ = 0.33 e Å^−3^
Δρ_min_ = −0.22 e Å^−3^

### 

Data collection: *SMART* (Bruker, 2007 ▶); cell refinement: *SAINT* (Bruker, 2007 ▶); data reduction: *SAINT*; program(s) used to solve structure: *SHELXTL* (Sheldrick, 2008 ▶); program(s) used to refine structure: *SHELXTL*; molecular graphics: *SHELXTL*, *OLEX2* (Dolomanov *et al*., 2009 ▶) and *DIAMOND* (Brandenburg, 1999 ▶); software used to prepare material for publication: *SHELXTL*, *modiCIFer* (Guzei, 2007 ▶) and *publCIF* (Westrip, 2009 ▶).

## Supplementary Material

Crystal structure: contains datablocks global, I. DOI: 10.1107/S1600536809040756/bv2130sup1.cif

Structure factors: contains datablocks I. DOI: 10.1107/S1600536809040756/bv2130Isup2.hkl

Additional supplementary materials:  crystallographic information; 3D view; checkCIF report

## Figures and Tables

**Fig. 1.**
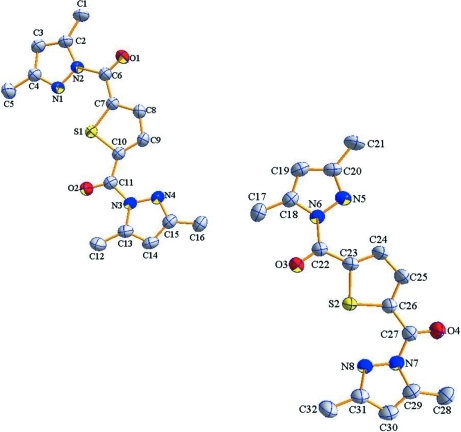
Molecular structure of (I). The thermal ellipsoids are shown at 50% probability level. All hydrogen atoms are ommitted for clarity.

**Fig. 2.**
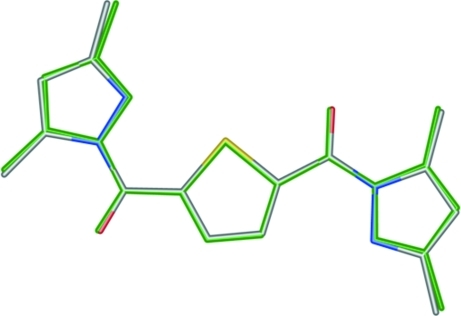
An overlap diagram of the two independent molecules of (I) in the asymmetric unit showing their differences.

## supplementary crystallographic information

### Comment

In the course of our studies toward effective polymerization catalysts we investigated Pd complexes with pyrazolyl derivatives as ligands. Benzene carbonyl (Guzei *et al.*, 2003) and pyridine carbonyl (Mohlala *et al.*, 2005) were used as linkers between two pyrazolyl units which served as ligands that readily form complexes with palladium. During the project development the title compound (I) was serendipitously isolated. Single crystals of (I) were obtained by slow evaporation of its dichloromethane:hexane (2:1) solution.

Compound (I) crystallizes with two symmetry independent half-molecules in the asymmetric unit. Each molecule lies in a crystallographic mirror plane. Compound (I) (Fig. 1) has typical bond distances and angles as confirmed by the *Mogul* structural check (Bruno *et al.*, 2002) and a comparison to 11 related compounds in the Cambridge Structural Database (Allen, 2002). The two molecules of compound (I) present in the asymmetric unit have essentially identical geometries as illustrated in the overlay diagram (Fig. 2).

In the crystal the molecules of (I) form sheets in the *a-c* plane which then form stacks along the *b* axis. The molecules within the sheets are joined by a weak C—H···O hydrogen bonding interaction, C8—H8···O4. The sheets are separated by a distance equal to the length of the *b* axis. The stacking of the sheets is aided by several weak π-π stacking interactions between atoms C3 and C6 (3.374 Å) and atoms C27 and C30 (3.390 Å). Two weak hydrogen bonding interactions, C21—H21C···O3 and C16—H16B···O2, of the type C—H···O also contribute to the stacking of the sheets.

### Experimental

3,5-Dimethylpyrazole (0.63 g, 6.76 mmol) and 2 ml of Et_3_N were added to a solution of 2,5-thiophenedicarbonyl dichloride (0.70 g, 3.34 mmol) in toluene (40 ml), and the resultant solution was refluxed 24 h. The reaction was filtered to remove the Et_3_NH^+^Cl^-^ by-product, and the solvent was evaporated from the filtrate to give a yellow residue. The yellow solid was purified by chromatography on silica gel using a dichloromethane:diethyl ether (8:1) mixture as eluent. Removal of the solvent from the eluent gave analytically pure product. Single crystals suitable for X-ray studies were obtained from dichloromethane:hexane(2:1) solution of (I). Yield: 0.85 g, 78%. ^1^H NMR (CDCl_3_): δ 8.24 (s, 2H, thiophene); 6.06 (s, 2H, 4-pz); 2.63 (s, 6H, 5-Mepz); 2.33 (s, 6H, 3-Mepz). ^13^C{^1^H} NMR: δ 160.6, 152.5, 145.1, 142.7, 135.8, 111.6, 14.4, 13.8. IR (nulo mull): µ(C=O) 1680 cm^-1^.

### Refinement

All H-atoms were placed in geometrically idealized locations with C—H distances of 0.96 Å to the primary and 0.93 Å to the aromatic carbon atoms. The H-atoms were refined as riding with thermal displacement coefficients *U*_iso_(H) = 1.5 times *U*_eq_(bearinng C atom). One hydrogen atom attached to carbon atoms C1, C5, C12, C16, C17, C21, C28, and C32 is equally disordered over two positions about the mirror plane.

### Crystal data

**Table:**

C_16_H_16_N_4_O_2_S	*F*(000) = 688
*M**_r_* = 328.39	*D*_x_ = 1.360 Mg m^−^^3^
Monoclinic, *P*2_1_/*m*	Mo *K*α radiation, λ = 0.71073 Å
Hall symbol: -P 2yb	Cell parameters from 999 reflections
*a* = 15.615 (3) Å	θ = 1.3–26.3°
*b* = 6.7153 (16) Å	µ = 0.22 mm^−^^1^
*c* = 16.803 (4) Å	*T* = 296 K
β = 114.452 (4)°	Block, colourless
*V* = 1603.9 (6) Å^3^	0.30 × 0.30 × 0.20 mm
*Z* = 4	

### Data collection

**Table:**

Bruker CCD 1000 area-detector diffractometer	3297 independent reflections
Radiation source: fine-focus sealed tube	2744 reflections with *I* > 2σ(*I*)
graphite	*R*_int_ = 0.017
0.30° ω scans	θ_max_ = 26.3°, θ_min_ = 1.3°
Absorption correction: multi-scan (*SADABS*; Bruker, 2007)	*h* = −18→7
*T*_min_ = 0.938, *T*_max_ = 0.958	*k* = −8→8
7557 measured reflections	*l* = −18→20

### Refinement

**Table:**

Refinement on *F*^2^	Secondary atom site location: difference Fourier map
Least-squares matrix: full	Hydrogen site location: inferred from neighbouring sites
*R*[*F*^2^ > 2σ(*F*^2^)] = 0.039	H-atom parameters constrained
*wR*(*F*^2^) = 0.112	*w* = 1/[σ^2^(*F*_o_^2^) + (0.0765*P*)^2^ + 0.2093*P*] where *P* = (*F*_o_^2^ + 2*F*_c_^2^)/3
*S* = 1.03	(Δ/σ)_max_ < 0.001
3297 reflections	Δρ_max_ = 0.33 e Å^−^^3^
286 parameters	Δρ_min_ = −0.22 e Å^−^^3^
0 restraints	Extinction correction: *SHELXTL* (Version 6.10; Sheldrick, 2008), Fc^*^=kFc[1+0.001xFc^2^λ^3^/sin(2θ)]^-1/4^
Primary atom site location: structure-invariant direct methods	Extinction coefficient: 0.0041 (8)

### Special details

**Table:**

Geometry. All e.s.d.'s (except the e.s.d. in the dihedral angle between two l.s. planes) are estimated using the full covariance matrix. The cell e.s.d.'s are taken into account individually in the estimation of e.s.d.'s in distances, angles and torsion angles; correlations between e.s.d.'s in cell parameters are only used when they are defined by crystal symmetry. An approximate (isotropic) treatment of cell e.s.d.'s is used for estimating e.s.d.'s involving l.s. planes.
Refinement. Refinement of *F*^2^ against ALL reflections. The weighted *R*-factor *wR* and goodness of fit *S* are based on *F*^2^, conventional *R*-factors *R* are based on *F*, with *F* set to zero for negative *F*^2^. The threshold expression of *F*^2^ > σ(*F*^2^) is used only for calculating *R*-factors(gt) *etc*. and is not relevant to the choice of reflections for refinement. *R*-factors based on *F*^2^ are statistically about twice as large as those based on *F*, and *R*- factors based on ALL data will be even larger.

### Fractional atomic coordinates and isotropic or equivalent isotropic displacement parameters (Å^2^)

**Table:**

	*x*	*y*	*z*	*U*_iso_*/*U*_eq_	Occ. (<1)
S1	0.76388 (4)	0.2500	−0.02885 (3)	0.04546 (18)	
S2	0.21011 (4)	0.7500	0.46771 (3)	0.04529 (18)	
O1	1.02198 (12)	0.2500	0.16162 (10)	0.0602 (5)	
O2	0.56735 (13)	0.2500	−0.11813 (11)	0.0656 (5)	
O3	0.32829 (12)	0.7500	0.37697 (10)	0.0598 (5)	
O4	0.12968 (14)	0.7500	0.66244 (11)	0.0687 (5)	
N1	0.91549 (13)	0.2500	−0.06725 (12)	0.0463 (5)	
N2	0.98581 (13)	0.2500	0.01605 (11)	0.0438 (4)	
N3	0.51710 (14)	0.2500	−0.01002 (13)	0.0491 (5)	
N4	0.53697 (15)	0.2500	0.07758 (13)	0.0538 (5)	
N5	0.53108 (14)	0.7500	0.57482 (12)	0.0504 (5)	
N6	0.47457 (13)	0.7500	0.48641 (12)	0.0454 (5)	
N7	0.03038 (14)	0.7500	0.51918 (12)	0.0472 (5)	
N8	0.02073 (14)	0.7500	0.43423 (13)	0.0499 (5)	
C1	1.16450 (17)	0.2500	0.09562 (16)	0.0545 (6)	
H1A	1.1746	0.3792	0.1224	0.082*	0.50
H1B	1.2154	0.2187	0.0798	0.082*	0.50
H1C	1.1616	0.1521	0.1361	0.082*	0.50
C2	1.07409 (16)	0.2500	0.01566 (15)	0.0440 (5)	
C3	1.05827 (17)	0.2500	−0.06987 (15)	0.0493 (6)	
H3	1.1037	0.2500	−0.0922	0.059*	
C4	0.95929 (17)	0.2500	−0.11908 (15)	0.0461 (5)	
C5	0.9038 (2)	0.2500	−0.21587 (16)	0.0638 (7)	
H5A	0.9079	0.1213	−0.2389	0.096*	0.50
H5B	0.9286	0.3490	−0.2418	0.096*	0.50
H5C	0.8391	0.2797	−0.2291	0.096*	0.50
C6	0.96182 (16)	0.2500	0.08757 (14)	0.0433 (5)	
C7	0.86051 (16)	0.2500	0.06999 (14)	0.0426 (5)	
C8	0.83320 (18)	0.2500	0.13777 (14)	0.0504 (6)	
H8	0.8757	0.2500	0.1962	0.060*	
C9	0.73637 (18)	0.2500	0.11117 (15)	0.0530 (6)	
H9	0.7073	0.2500	0.1496	0.064*	
C10	0.68842 (17)	0.2500	0.02215 (14)	0.0446 (5)	
C11	0.58809 (18)	0.2500	−0.04065 (15)	0.0498 (6)	
C12	0.3760 (2)	0.2500	−0.15831 (18)	0.0785 (9)	
H12A	0.4051	0.3497	−0.1799	0.118*	0.50
H12B	0.3102	0.2787	−0.1783	0.118*	0.50
H12C	0.3839	0.1216	−0.1795	0.118*	0.50
C13	0.42081 (18)	0.2500	−0.06136 (16)	0.0544 (6)	
C14	0.38063 (19)	0.2500	−0.00366 (18)	0.0594 (7)	
H14	0.3165	0.2500	−0.0172	0.071*	
C15	0.45420 (19)	0.2500	0.08064 (17)	0.0553 (6)	
C16	0.4463 (2)	0.2500	0.16601 (19)	0.0737 (9)	
H16A	0.3920	0.1744	0.1608	0.111*	0.50
H16B	0.4401	0.3844	0.1823	0.111*	0.50
H16C	0.5017	0.1912	0.2100	0.111*	0.50
C17	0.4882 (2)	0.7500	0.34029 (17)	0.0690 (8)	
H17A	0.4527	0.8697	0.3183	0.103*	0.50
H17B	0.4479	0.6365	0.3181	0.103*	0.50
H17C	0.5387	0.7438	0.3218	0.103*	0.50
C18	0.52755 (18)	0.7500	0.43745 (16)	0.0498 (6)	
C19	0.61859 (18)	0.7500	0.49724 (17)	0.0552 (6)	
H19	0.6717	0.7500	0.4853	0.066*	
C20	0.61758 (17)	0.7500	0.58059 (17)	0.0511 (6)	
C21	0.69948 (19)	0.7500	0.66823 (18)	0.0696 (8)	
H21A	0.6806	0.8065	0.7109	0.104*	0.50
H21B	0.7497	0.8277	0.6653	0.104*	0.50
H21C	0.7206	0.6158	0.6845	0.104*	0.50
C22	0.37624 (16)	0.7500	0.45456 (14)	0.0448 (5)	
C23	0.33142 (16)	0.7500	0.51683 (14)	0.0447 (5)	
C24	0.36440 (18)	0.7500	0.60527 (16)	0.0651 (8)	
H24	0.4280	0.7500	0.6427	0.078*	
C25	0.29274 (19)	0.7500	0.63368 (16)	0.0659 (8)	
H25	0.3038	0.7500	0.6925	0.079*	
C26	0.20405 (17)	0.7500	0.56718 (14)	0.0469 (5)	
C27	0.12019 (18)	0.7500	0.58736 (15)	0.0488 (6)	
C28	−0.0696 (2)	0.7500	0.60687 (19)	0.0652 (7)	
H28A	−0.0307	0.8509	0.6454	0.098*	0.50
H28B	−0.0525	0.6222	0.6346	0.098*	0.50
H28C	−0.1344	0.7769	0.5939	0.098*	0.50
C29	−0.05587 (18)	0.7500	0.52427 (17)	0.0515 (6)	
C30	−0.12032 (19)	0.7500	0.44038 (18)	0.0578 (6)	
H30	−0.1853	0.7500	0.4215	0.069*	
C31	−0.07062 (18)	0.7500	0.38631 (17)	0.0536 (6)	
C32	−0.1090 (2)	0.7500	0.28947 (18)	0.0739 (8)	
H32A	−0.0600	0.7157	0.2714	0.111*	0.50
H32B	−0.1329	0.8800	0.2678	0.111*	0.50
H32C	−0.1590	0.6543	0.2665	0.111*	0.50

### Atomic displacement parameters (Å^2^)

**Table:**

	*U*^11^	*U*^22^	*U*^33^	*U*^12^	*U*^13^	*U*^23^
S1	0.0355 (3)	0.0652 (4)	0.0380 (3)	0.000	0.0176 (2)	0.000
S2	0.0319 (3)	0.0649 (4)	0.0372 (3)	0.000	0.0124 (2)	0.000
O1	0.0441 (10)	0.0916 (13)	0.0403 (9)	0.000	0.0128 (7)	0.000
O2	0.0464 (11)	0.1080 (15)	0.0426 (9)	0.000	0.0186 (8)	0.000
O3	0.0430 (10)	0.0934 (13)	0.0402 (9)	0.000	0.0144 (7)	0.000
O4	0.0543 (12)	0.1112 (15)	0.0454 (9)	0.000	0.0256 (8)	0.000
N1	0.0349 (11)	0.0628 (12)	0.0396 (10)	0.000	0.0138 (8)	0.000
N2	0.0351 (10)	0.0561 (11)	0.0392 (10)	0.000	0.0143 (8)	0.000
N3	0.0364 (11)	0.0665 (13)	0.0462 (10)	0.000	0.0188 (8)	0.000
N4	0.0417 (12)	0.0752 (14)	0.0492 (11)	0.000	0.0233 (9)	0.000
N5	0.0370 (11)	0.0640 (12)	0.0449 (10)	0.000	0.0117 (8)	0.000
N6	0.0381 (11)	0.0546 (11)	0.0438 (10)	0.000	0.0173 (8)	0.000
N7	0.0391 (11)	0.0586 (12)	0.0472 (11)	0.000	0.0211 (9)	0.000
N8	0.0360 (11)	0.0690 (13)	0.0442 (10)	0.000	0.0162 (8)	0.000
C1	0.0325 (13)	0.0699 (16)	0.0567 (14)	0.000	0.0140 (10)	0.000
C2	0.0325 (12)	0.0491 (12)	0.0507 (13)	0.000	0.0174 (9)	0.000
C3	0.0399 (13)	0.0622 (14)	0.0517 (13)	0.000	0.0249 (11)	0.000
C4	0.0417 (13)	0.0548 (13)	0.0447 (12)	0.000	0.0209 (10)	0.000
C5	0.0570 (17)	0.092 (2)	0.0424 (13)	0.000	0.0205 (12)	0.000
C6	0.0388 (13)	0.0516 (12)	0.0401 (11)	0.000	0.0171 (9)	0.000
C7	0.0392 (12)	0.0495 (12)	0.0391 (11)	0.000	0.0163 (9)	0.000
C8	0.0455 (14)	0.0685 (15)	0.0383 (11)	0.000	0.0186 (10)	0.000
C9	0.0472 (15)	0.0761 (16)	0.0427 (12)	0.000	0.0255 (10)	0.000
C10	0.0400 (13)	0.0537 (13)	0.0447 (12)	0.000	0.0223 (10)	0.000
C11	0.0413 (14)	0.0624 (14)	0.0480 (13)	0.000	0.0210 (10)	0.000
C12	0.0435 (16)	0.126 (3)	0.0563 (16)	0.000	0.0109 (12)	0.000
C13	0.0384 (14)	0.0671 (16)	0.0564 (14)	0.000	0.0184 (11)	0.000
C14	0.0359 (14)	0.0757 (17)	0.0704 (17)	0.000	0.0256 (12)	0.000
C15	0.0474 (16)	0.0678 (16)	0.0581 (15)	0.000	0.0294 (12)	0.000
C16	0.0614 (19)	0.110 (2)	0.0636 (17)	0.000	0.0393 (15)	0.000
C17	0.0647 (19)	0.097 (2)	0.0557 (15)	0.000	0.0353 (14)	0.000
C18	0.0462 (14)	0.0540 (13)	0.0554 (14)	0.000	0.0273 (11)	0.000
C19	0.0392 (14)	0.0632 (15)	0.0684 (16)	0.000	0.0275 (12)	0.000
C20	0.0337 (13)	0.0561 (14)	0.0593 (15)	0.000	0.0151 (11)	0.000
C21	0.0384 (15)	0.091 (2)	0.0659 (17)	0.000	0.0081 (12)	0.000
C22	0.0381 (13)	0.0532 (13)	0.0427 (12)	0.000	0.0161 (10)	0.000
C23	0.0316 (12)	0.0543 (13)	0.0433 (12)	0.000	0.0107 (9)	0.000
C24	0.0347 (13)	0.115 (2)	0.0405 (12)	0.000	0.0104 (10)	0.000
C25	0.0417 (15)	0.117 (2)	0.0370 (12)	0.000	0.0139 (10)	0.000
C26	0.0399 (13)	0.0590 (14)	0.0416 (12)	0.000	0.0166 (10)	0.000
C27	0.0425 (14)	0.0605 (14)	0.0445 (12)	0.000	0.0189 (10)	0.000
C28	0.0601 (18)	0.0769 (18)	0.0756 (18)	0.000	0.0452 (15)	0.000
C29	0.0419 (14)	0.0557 (14)	0.0645 (15)	0.000	0.0298 (12)	0.000
C30	0.0362 (14)	0.0664 (16)	0.0729 (17)	0.000	0.0247 (12)	0.000
C31	0.0385 (14)	0.0618 (14)	0.0573 (14)	0.000	0.0166 (11)	0.000
C32	0.0474 (16)	0.109 (2)	0.0545 (15)	0.000	0.0099 (12)	0.000

### Geometric parameters (Å, °)

**Table:**

S1—C10	1.720 (2)	C10—C11	1.483 (3)
S1—C7	1.721 (2)	C12—C13	1.483 (4)
S2—C26	1.713 (2)	C12—H12A	0.9600
S2—C23	1.725 (2)	C12—H12B	0.9600
O1—C6	1.209 (3)	C12—H12C	0.9600
O2—C11	1.204 (3)	C13—C14	1.355 (4)
O3—C22	1.205 (3)	C14—C15	1.407 (4)
O4—C27	1.208 (3)	C14—H14	0.9300
N1—C4	1.311 (3)	C15—C16	1.490 (3)
N1—N2	1.376 (2)	C16—H16A	0.9600
N2—C2	1.381 (3)	C16—H16B	0.9600
N2—C6	1.399 (3)	C16—H16C	0.9600
N3—N4	1.373 (3)	C17—C18	1.487 (3)
N3—C13	1.390 (3)	C17—H17A	0.9600
N3—C11	1.402 (3)	C17—H17B	0.9600
N4—C15	1.315 (3)	C17—H17C	0.9600
N5—C20	1.314 (3)	C18—C19	1.359 (4)
N5—N6	1.379 (3)	C19—C20	1.407 (4)
N6—C18	1.387 (3)	C19—H19	0.9300
N6—C22	1.401 (3)	C20—C21	1.498 (3)
N7—N8	1.372 (3)	C21—H21A	0.9600
N7—C29	1.384 (3)	C21—H21B	0.9600
N7—C27	1.396 (3)	C21—H21C	0.9600
N8—C31	1.316 (3)	C22—C23	1.480 (3)
C1—C2	1.493 (3)	C23—C24	1.356 (3)
C1—H1A	0.9600	C24—C25	1.387 (4)
C1—H1B	0.9600	C24—H24	0.9300
C1—H1C	0.9600	C25—C26	1.373 (3)
C2—C3	1.354 (3)	C25—H25	0.9300
C3—C4	1.419 (3)	C26—C27	1.482 (3)
C3—H3	0.9300	C28—C29	1.489 (3)
C4—C5	1.493 (3)	C28—H28A	0.9600
C5—H5A	0.9600	C28—H28B	0.9600
C5—H5B	0.9600	C28—H28C	0.9600
C5—H5C	0.9600	C29—C30	1.351 (4)
C6—C7	1.484 (3)	C30—C31	1.418 (4)
C7—C8	1.372 (3)	C30—H30	0.9300
C8—C9	1.388 (4)	C31—C32	1.483 (4)
C8—H8	0.9300	C32—H32A	0.9600
C9—C10	1.368 (3)	C32—H32B	0.9600
C9—H9	0.9300	C32—H32C	0.9600
			
C10—S1—C7	91.54 (11)	N4—C15—C14	111.5 (2)
C26—S2—C23	91.51 (11)	N4—C15—C16	120.8 (3)
C4—N1—N2	105.05 (18)	C14—C15—C16	127.7 (3)
N1—N2—C2	111.90 (17)	C15—C16—H16A	109.5
N1—N2—C6	119.30 (18)	C15—C16—H16B	109.5
C2—N2—C6	128.8 (2)	H16A—C16—H16B	109.5
N4—N3—C13	111.83 (18)	C15—C16—H16C	109.5
N4—N3—C11	122.10 (19)	H16A—C16—H16C	109.5
C13—N3—C11	126.1 (2)	H16B—C16—H16C	109.5
C15—N4—N3	104.6 (2)	C18—C17—H17A	109.5
C20—N5—N6	105.0 (2)	C18—C17—H17B	109.5
N5—N6—C18	111.48 (19)	H17A—C17—H17B	109.5
N5—N6—C22	121.53 (19)	C18—C17—H17C	109.5
C18—N6—C22	126.98 (19)	H17A—C17—H17C	109.5
N8—N7—C29	111.94 (19)	H17B—C17—H17C	109.5
N8—N7—C27	119.62 (19)	C19—C18—N6	105.0 (2)
C29—N7—C27	128.4 (2)	C19—C18—C17	129.9 (2)
C31—N8—N7	105.1 (2)	N6—C18—C17	125.1 (2)
C2—C1—H1A	109.5	C18—C19—C20	107.3 (2)
C2—C1—H1B	109.5	C18—C19—H19	126.4
H1A—C1—H1B	109.5	C20—C19—H19	126.4
C2—C1—H1C	109.5	N5—C20—C19	111.2 (2)
H1A—C1—H1C	109.5	N5—C20—C21	120.4 (2)
H1B—C1—H1C	109.5	C19—C20—C21	128.4 (2)
C3—C2—N2	105.1 (2)	C20—C21—H21A	109.5
C3—C2—C1	130.1 (2)	C20—C21—H21B	109.5
N2—C2—C1	124.7 (2)	H21A—C21—H21B	109.5
C2—C3—C4	107.1 (2)	C20—C21—H21C	109.5
C2—C3—H3	126.4	H21A—C21—H21C	109.5
C4—C3—H3	126.4	H21B—C21—H21C	109.5
N1—C4—C3	110.8 (2)	O3—C22—N6	120.3 (2)
N1—C4—C5	119.7 (2)	O3—C22—C23	120.1 (2)
C3—C4—C5	129.5 (2)	N6—C22—C23	119.61 (19)
C4—C5—H5A	109.5	C24—C23—C22	134.3 (2)
C4—C5—H5B	109.5	C24—C23—S2	111.59 (18)
H5A—C5—H5B	109.5	C22—C23—S2	114.13 (16)
C4—C5—H5C	109.5	C23—C24—C25	112.5 (2)
H5A—C5—H5C	109.5	C23—C24—H24	123.8
H5B—C5—H5C	109.5	C25—C24—H24	123.8
O1—C6—N2	120.9 (2)	C26—C25—C24	113.9 (2)
O1—C6—C7	121.0 (2)	C26—C25—H25	123.0
N2—C6—C7	118.12 (19)	C24—C25—H25	123.0
C8—C7—C6	120.5 (2)	C25—C26—C27	120.2 (2)
C8—C7—S1	110.59 (18)	C25—C26—S2	110.47 (18)
C6—C7—S1	128.96 (17)	C27—C26—S2	129.35 (18)
C7—C8—C9	113.8 (2)	O4—C27—N7	120.3 (2)
C7—C8—H8	123.1	O4—C27—C26	120.1 (2)
C9—C8—H8	123.1	N7—C27—C26	119.7 (2)
C10—C9—C8	112.5 (2)	C29—C28—H28A	109.5
C10—C9—H9	123.8	C29—C28—H28B	109.5
C8—C9—H9	123.8	H28A—C28—H28B	109.5
C9—C10—C11	135.8 (2)	C29—C28—H28C	109.5
C9—C10—S1	111.54 (19)	H28A—C28—H28C	109.5
C11—C10—S1	112.66 (16)	H28B—C28—H28C	109.5
O2—C11—N3	119.8 (2)	C30—C29—N7	105.0 (2)
O2—C11—C10	120.1 (2)	C30—C29—C28	129.8 (3)
N3—C11—C10	120.1 (2)	N7—C29—C28	125.2 (2)
C13—C12—H12A	109.5	C29—C30—C31	107.4 (2)
C13—C12—H12B	109.5	C29—C30—H30	126.3
H12A—C12—H12B	109.5	C31—C30—H30	126.3
C13—C12—H12C	109.5	N8—C31—C30	110.5 (2)
H12A—C12—H12C	109.5	N8—C31—C32	121.0 (2)
H12B—C12—H12C	109.5	C30—C31—C32	128.5 (2)
C14—C13—N3	105.0 (2)	C31—C32—H32A	109.5
C14—C13—C12	129.7 (3)	C31—C32—H32B	109.5
N3—C13—C12	125.4 (2)	H32A—C32—H32B	109.5
C13—C14—C15	107.1 (2)	C31—C32—H32C	109.5
C13—C14—H14	126.5	H32A—C32—H32C	109.5
C15—C14—H14	126.5	H32B—C32—H32C	109.5
			
C4—N1—N2—C2	0.0	N3—N4—C15—C14	0.0
C4—N1—N2—C6	180.0	N3—N4—C15—C16	180.0
C13—N3—N4—C15	0.0	C13—C14—C15—N4	0.0
C11—N3—N4—C15	180.0	C13—C14—C15—C16	180.0
C20—N5—N6—C18	0.0	N5—N6—C18—C19	0.000 (1)
C20—N5—N6—C22	180.0	C22—N6—C18—C19	180.0
C29—N7—N8—C31	0.0	N5—N6—C18—C17	180.0
C27—N7—N8—C31	180.0	C22—N6—C18—C17	0.000 (1)
N1—N2—C2—C3	0.0	N6—C18—C19—C20	0.0
C6—N2—C2—C3	180.0	C17—C18—C19—C20	180.0
N1—N2—C2—C1	180.0	N6—N5—C20—C19	0.0
C6—N2—C2—C1	0.0	N6—N5—C20—C21	180.0
N2—C2—C3—C4	0.0	C18—C19—C20—N5	0.0
C1—C2—C3—C4	180.0	C18—C19—C20—C21	180.0
N2—N1—C4—C3	0.0	N5—N6—C22—O3	180.0
N2—N1—C4—C5	180.0	C18—N6—C22—O3	0.000 (1)
C2—C3—C4—N1	0.0	N5—N6—C22—C23	0.000 (1)
C2—C3—C4—C5	180.0	C18—N6—C22—C23	180.0
N1—N2—C6—O1	180.0	O3—C22—C23—C24	180.0
C2—N2—C6—O1	0.0	N6—C22—C23—C24	0.000 (1)
N1—N2—C6—C7	0.0	O3—C22—C23—S2	0.000 (1)
C2—N2—C6—C7	180.0	N6—C22—C23—S2	180.0
O1—C6—C7—C8	0.0	C26—S2—C23—C24	0.0
N2—C6—C7—C8	180.0	C26—S2—C23—C22	180.0
O1—C6—C7—S1	180.0	C22—C23—C24—C25	180.0
N2—C6—C7—S1	0.0	S2—C23—C24—C25	0.0
C10—S1—C7—C8	0.0	C23—C24—C25—C26	0.0
C10—S1—C7—C6	180.0	C24—C25—C26—C27	180.0
C6—C7—C8—C9	180.0	C24—C25—C26—S2	0.0
S1—C7—C8—C9	0.0	C23—S2—C26—C25	0.0
C7—C8—C9—C10	0.0	C23—S2—C26—C27	180.0
C8—C9—C10—C11	180.0	N8—N7—C27—O4	180.0
C8—C9—C10—S1	0.0	C29—N7—C27—O4	0.0
C7—S1—C10—C9	0.0	N8—N7—C27—C26	0.0
C7—S1—C10—C11	180.0	C29—N7—C27—C26	180.0
N4—N3—C11—O2	180.0	C25—C26—C27—O4	0.000 (1)
C13—N3—C11—O2	0.0	S2—C26—C27—O4	180.0
N4—N3—C11—C10	0.0	C25—C26—C27—N7	180.0
C13—N3—C11—C10	180.0	S2—C26—C27—N7	0.0
C9—C10—C11—O2	180.0	N8—N7—C29—C30	0.0
S1—C10—C11—O2	0.0	C27—N7—C29—C30	180.0
C9—C10—C11—N3	0.0	N8—N7—C29—C28	180.0
S1—C10—C11—N3	180.0	C27—N7—C29—C28	0.000 (1)
N4—N3—C13—C14	0.0	N7—C29—C30—C31	0.0
C11—N3—C13—C14	180.0	C28—C29—C30—C31	180.0
N4—N3—C13—C12	180.0	N7—N8—C31—C30	0.0
C11—N3—C13—C12	0.0	N7—N8—C31—C32	180.0
N3—C13—C14—C15	0.0	C29—C30—C31—N8	0.000 (1)
C12—C13—C14—C15	180.0	C29—C30—C31—C32	180.0
